# An Inquiry, Critical and Experimental, into the Pathology of Fever

**Published:** 1853-10

**Authors:** N. S. Davis

**Affiliations:** Professor of Pathology, Practice of Medicine, and Clinical Medicine, in Rush Medical College; and one of the Physicians to the Ill. Gen. Hospital


*An Inquiry, Critical and Experimental, into the Pathology of Fever.* By N. S. Davis M. D. Professor of Pathology, Practice of Medicine, and Clinical Medicine, in Rush Medical College; and one of the Physicians to the Ill. Gen. Hospital.

[Continued from Page 320.]

### Chapter Second.

### DIAGNOSIS AND VARIETIES OF FEVER.

---

So long as the pathology of fevers remains unsettled and a subject of controversy, there must also be much diversity of opinion, if not absolute confusion, in regard to the varieties and diagnostic phenomena on which the several classifications are based.

Thus, Dr. Stokes, of Dublin, tells us to "weigh the matter calmly, and I think you will be disposed to agree with me, that *fever*, in its origin, implies no tangible condition of the system, and that we know it only as consisting of a group of phenomena, varying as to their cause, seat, effect, and duration."

Dr. Bartlett, in his valuable treatise on fevers, observes: "There is *no such individual disease*, as that which has always been expressed, and which is still expressed by the term *fever*. * * * * There are many separate diseases to which this generic name is properly enough applied, on account of certain general analogies which exist between them. * * * * *The word fever, when used as it commonly is, to designate a disease, has no intelligible signification.* It is wholly a creature of the fancy; the offspring of a false generalization, and of a spurious philosophy."

On the same subject Dr. Southwood Smith observes: "Something there is, however, which, amidst this astonishing diversity, preserves the identity of the disease so completely and so obviously, that there never has existed any dispute about that identity, under any aspect which it has hitherto been observed to assume, so that all physicians, without exception, unhesitatingly accord the name of *fever* to the mildest form of the common fever of this country, to the yellow fever of the West Indies, and to the plague of Constantinople and of Egypt."

Dr. Thomas Watson says: "Although *fever is*, as I have stated, a *specific disease*, it assumes diverse forms, and so dissimi-

Reel 178-10-5 CONT

lar are some of its phases, that they might seem to belong to totally different maladies."

Dr. Benj. Rush has said, that it is "not more improper to say that men are of different species, because some are tall and others short, or because some are short and others long-lived, *than that fevers are of different species*, because they vary in their symptoms and duration."

Again, Dr. S. H. Dickson, of Charleston, in a recent interesting essay, remarks: "We cannot for a moment admit the truth of the specious doctrine, that fever, or, as it is imaginatively termed, the '*febrile element*,' is a unit, identical in all the forms of that protean malady. On the contrary, we are prepared to maintain that, from the great diversity of specific causes of fever, a great diversity of specific effects must necessarily result." And yet, as the reader has already seen by a quotation at the beginning of this lengthened essay, the eminent Dr. Copeland says, "*fever is but one disease.*"

We might add to the foregoing, many more quotations from distinguished members of the profession, touching the same subject, but these are sufficient to show that there is, not only no division or classification of fevers generally adopted, but there is no admitted basis on which a judicious classification can be founded.

Some evidently regard all fevers, however diverse in their general phenomena, as having essentially a common pathological element or starting point, which constitutes a bond of union, and in some sense reduces them to a unit. This seems to be the idea entertained by Rush, Copeland, Southwood Smith, Watson, Paine, and others; but scarcely two of these agree in their views in regard to what constitutes this *unity*, this common febrile element, or essential starting point. Some of them place it in the depressing action of the exciting cause or causes on the *nervous system*, others regard it invariably as an exaltation of certain elementary properties of the solids, while others still frankly acknowledge their entire ignorance concerning it. On the other hand, those who, like Drs. Stokes, Bartlett, and Dickson, deny the unity of fevers, and claim that the different varieties are specifically separate and distinct diseases, are no better agreed in regard either to the number of specific fevers, or the particular basis on which their separation should rest. Thus, Dr. Dickson says: "Specific difference in causative or generative agency will give an absolute specific difference in results. If, for example, some fevers are contagious, and others not at all, this single fact must constitute solely and of itself an entire essentiality of diagnosis." This would seem to indicate a classification, founded on the nature of the exciting cause. Dr Bartlett, following the example of Louis, Chomel, and Jackson, attempts to base his classification partly on the phenomena of the diseases during life, and partly on the effects or post-mortem appearances. The periodical class are separated from all others on account of their periodicity and the supposed peculiarity of the exciting cause. But the distinction between typhoid and typhus fevers, is made to rest more on the morbid anatomy, than the symptoms during life. Wherever so great diversity of opinion exists among men, eminent alike for their science and their practical experience, we may safely infer that there is either no clear and well defined starting point to the subject under investigation, or that they have not occupied any common point of observation in relation to it. Thus, when Dr. Copeland asserts that "fever is but *one* disease," and Dr. Bartlett declares that "there is no such individual disease as that which has always been expressed by the term *fever*," it is clearly apparent, that the two writers are viewing the same subject from entirely different points of observation.

The one is evidently looking at the initial active phenomena of a class of diseases, as they exist in the perverted action of the same functions and vital properties, regardless of diversity in *causation* or ultimate anatomical results. Hence he finds no difficulty in tracing these phenomena, however diverse in some respects, to disturbance or perversion of the same primary functions and properties, thereby finding a bond of union on which to base the declaration that fever is but *one disease*. But the other, with a mind early captivated by the interesting researches of M. Louis and other French pathologists, is looking much more intently at the specific differences in causation and ultimate morbid changes, than at the immediate initial febrile phenomena. If I might choose a figure for illustration, I should represent Dr. Copeland as standing at the circumference of a class of diseases, looking for some central point towards which the essential active phenomena of each member of the class converge; while Dr. Bartlett takes his station in the centre, and looks in one direction at the specifically distinct exciting causes, and in the other at the equally different post-mortem results. Hence it is not strange that the one should affirm, and the other deny the *unity* of the class. What is true of the two distinguished writers just named, is equally true of the several classes of medical men which they represent. But after making all the allowance that a just and liberal criticism will permit, for differences in mental position and direction of inquiry, there remains so much contrariety of opinion, that the inexperienced learner is quite as likely to become confused, as enlightened by an extensive study of medical writings on the subject under consideration.

We cannot, therefore, proceed a single step in the work of classifying fevers, and pointing out their diagnostic symptoms, without first presenting a definite basis for such arrangement as we propose to make. But what shall constitute this basis?

Shall we follow the hint given us by Dr. Dickson, when he asserts that "specific difference in causative or generative agency will give an absolutely specific difference in results," and found a classification on the exciting cause of fever? If it were true that every specific cause, capable of exciting fever, actually gave rise to an equally specific and well defined disease, it would be easy to arrange or classify them, although the subdivisions would even then be inconveniently numerous.

But Dr. Dickson himself does not claim this when he admits that "the effect must always be precisely relevant to the cause, *unless the latter undergoes modification, transformation, or substitution.*" I think that a careful examination will show these modifications or transformations to be so numerous as to destroy the practical value of the rule laid down in reference to cause and effect. That an insufficient supply of food, coupled with exposure to a cold and damp atmosphere, has frequently given rise to severe and fatal cases of typhus, very few will deny. That the same form of disease is caused still more certainly by confinement in an atmosphere rendered impure by an accumulation of animal excretions and inhalations is equally obvious. And yet, is there not a specific difference between cold coupled with hunger, and the foul air of an ill-ventilated ship? One of the most characteristic cases of *typhoid* fever which has come under my observation during the present season, was very obviously induced by frequent and protracted bathing in the lake, coupled with over exertion in swimming. Another well marked case of the same variety of fever, was caused by the combined influence of protracted wakefulness and anxiety caused by the sickness of a friend. Indeed, there is no fact more familiar to the profession, than that *dissimilar* causes often give rise to cases of disease, involving the same functions and organs, and identical in their form. This fact presents an insuperable barrier to any detailed classification founded on the nature of the exciting causes. Indeed, the whole subject of the causation of *fevers* is still involved in doubt and controversy. One class of writers continually speak of the *poison* of typhus and the *poison* of typhoid fever, as though these varieties of disease were dependent generally, if not invariably, on some *specific* and definite *poison*. Thus, Dr. Bartlett tells us, that "*the poison* of typhus fever is generated in a stagnant and depraved atmosphere, rank with the thick corruptions of concentrated emanations from the living human body; *the poison* of typhoid fever, like that of epidemic cholera, and like that of scarlatina, comes we know not whence; it is generated as readily amidst cleanliness and purity, as amidst filth; and it floats as freely in the fresh breezes under the open sky, as in the close and stagnant atmospheres of crowded cabins and lanes."

It is not my purpose to question the correctness of these assertions in this place, (that will be done elsewhere,) but simply to note the language in reference to the cause—"*the poison*;" certainly implying the existence of a single morbific agent, specific in its nature and determining by its action a specific form of fever. So, too, Dr. Watson remarks, "it is of great importance to hold correct notions as to the exciting cause of continued fever, respecting which there has been and still is, a perplexing contrariety of opinion among medical men. You are aware, from what has already been said, that I consider the disorder to originate in an *animal poison*, &c."

This language is explicit, and implies an unequivocal belief in the existence of a specific poison as the cause of fever. But even this class of writers are not agreed in reference to the question whether each depends on a poison peculiar to itself. The language already quoted, shows, that Dr. Bartlett claims a separate and distinct poison for typhus and typhoid fevers no less positively, than for the periodical class. On the other hand, Drs. Smith, Watson, and others make no definite distinction between typhus and typhoid diseases, and uniformly speak of but one fever poison as the cause of both. But there are still others of large practical experience, especially among American physicians in the southern and western states, who boldly claim that both of the varieties of continued fever, depend on some modification of the same malarial poison, which generates intermittents and remittents. And there are not wanting still others, deservedly eminent in the profession, who deny the existence of any specific fever poison whatever.

It is plain, that in the midst of so much uncertainty, and such contrariety of views, concerning what constitutes the efficient cause or causes of fevers, it would be futile to attempt to base any classification of these diseases on the nature of their exciting causes. If it were granted, however, that the causes of fevers were definitely known, any arrangement founded on them would possess very little practical value, for the simple reason, that causes essentially distinct, and arising out of circumstances very diverse, are known to give rise to febrile phenomena, identical in all their general and final results. By keeping this fact in view, we may reconcile much that appears contradictory in relation to the causation of fevers, without impeaching either the integrity or the acuteness of observation, of those whose written opinions stand so nearly antagonistic to each other.

Before prooceeding, however, let us enquire whether the reason given, the fact assumed, is itself true?

Will diverse causes develop the same febrile condition, presenting the same tendencies and ultimate results? In answering this question let us refer to that form of continued fever usually denominated *typhoid*, which is generally conceded to present a characteristic group of symptoms, and a pretty uniform tendency to a definite result, viz.: ulceration of the mucous membrane of the ilium. Dr. Bartlett says, "the only causes of typhoid fever, the influence of which has been at all positively and accurately ascertained, are these three, to wit: "age, recent residence in a given place, and contagion."

Dr. Watson admits but one efficient cause of continued fever, which he styles an *animal poison*. All the other circumstances which seem to exert an influence, such as age, locality, &c., he ranks as predisposing causes merely. To maintain this opinion, however, Dr. Watson, and all others who favor it, are compelled to assume, that the poison is self propagated and very widely, if not universally diffused; and yet, that its activity is so completely under the control of what they call *predisposing* causes, such as age, locality, recency of residence, etc., as to render it inoperative at one time and unusually virulent at another. Thus, with the exception of Dr. Bartlett, all writers admit that the disease is most prevalent in the midst of poverty, impure air, over-crowded apartments, and damp dwellings—that even the sick, when placed in spacious apartments, kept clean and well supplied with dry, pure air, are seldom capable of propagating the disease to others around them. So true is this, that every intelligent hospital physician knows full well the difference between spacious, clean, and well ventilated wards for fever patients, and such as are close, uncleanly, and crowded. In the first the nurses, physicians, and visitors, walk with comparative safety; while in the latter, not only the ratio of deaths among the patients is greatly increased, but the attendants perform their duties at the peril of their own safety. No fact in the history of disease is better established than this. Hence Dr. Watson says: "It is moreover found, that when persons, ill of fever, are taken away from their own close crowded houses, and when means of purification are employed, the fever ceases to spread in these houses, etc."

If it is true that, what are styled predisposing causes, determine in so marked a degree both the prevalence of the disease and its propagation; which is most in accordance with sound philosophy and rational induction, to attribute the disease to an unknown intangible agent floating everywhere, but entirely harmless in one place, and extremely active in another, and yet subject to no known or uniform laws; or to attribute it to those circumstances that are tangible, and the influence of which can be definitely appreciated. Within the last few weeks I have had under my care several well marked cases of typhoid fever both in and out of the hospital. Among these was a young man who came from the New England States to this city about six months since. He resided in one of the best families in the city, occupied during the day in spacious and airy apartments, and sleeping in a clean, dry, and well ventilated chamber. He continued to enjoy good health until the early part of August, when he adopted the practice of frequent bathing in the Lake. Being an excellent swimmer he carried the practice to an extent undoubtedly injurious, going in late in the evening and continuing in the water under active physical exertion for a considerable time. In a short time he began to complain of dulness, want of physical elasticity and vigor. This increased day by day until at the end of a week he was unable to continue his employment. He now had fever, headache, wandering pains in his back and limbs, tongue covered with a dirty white fur, little appetite, bowels irregular, not moving frequent but discharges liquid, pulse quick, and skin dry. All the symptoms were aggravated in the afternoon and evening.

He now consulted a physician who, viewing it as a "bilious attack," gave him a dose of physic, and followed it by moderate doses of quinine, which he continued to take for four or five days.

At the end of that time he came directly under my observation. His cheeks were slightly flushed; his eyes heavy and watery; his skin dry and above the natural temperature; his lips, mouth and teeth dry; tongue dry, smooth, and redder than natural; pulse 100 per minute; alvine evacuations fluid, dark brown, and occurring three or four times in 24 hours; abdomen moderately distended and tympanitic; intellect dull with slight wandering at night; complains of no definite pain, but feels dull, heavy, and indisposed to make any exertion, either mental or physical; and there were traces of a few rose colored spots over the anterior part of the body. The patient continued on with all the usual phenomena of typhoid fever, and slowly convalesced in about 10 days after he came under my care. Although surrounded by a large family, embracing persons of various ages from one year up to forty-five or fifty, none of them contracted the disease or suffered any inconvenience from his presence.

About the same time I was called to see a young lady, aged about 18 years, and found her with all the well marked symptoms of ordinary remittent fever of an active grade. The remissions and exacerbations, with all the characteristics of that type of fever, were strongly marked. I directed a few grains of sub. murias hydrargyri with dover's powder during the exacerbation, to be followed by sulphate of quinine in five gr. doses during the remission. They appeared to act kindly, and in four or five days she was entirely convalescent and able to rise from her bed, with some appetite, and a very natural condition of the alvine evacuations. Before recovering her strength, however, she imprudently ventured out and spent the evening with some friends, and indulged moderately in the use of such refreshments as were provided for the occasion. The next day she felt dull and heavy, with a little disturbance of her bowels. Her indisposition increased rather gradually for four or five days, at the end of which time I was again called in. I found her sitting in a chair, with a dull haggard expression of countenance; a deep red flush on her cheeks; her skin dry, harsh, and hot; pulse 102 per minute, and soft; lips, teeth, and mouth dry; tongue dry, red, and fissured; urine scanty and high colored; abdomen full and tympanitic, with tenderness on firm pressure; fecal discharges every six or eight hours, liquid, dark brown, and very offensive, dulness of intellect, with decided wandering delirium at times; much thirst; no appetite; and much complaint of dull pain in her head. From this time on her case retained all the features of the most severe typhoid fever, and terminated fatally in about three weeks. During her sickness she occupied a low chamber in a house filled with boarders, and her own room was often filled with an imprudent number of friends. About one week before her death, her sister, who was the mother of a child six months old, and spent most of the time in the room taking care of her, was taken sick with well marked symptoms of the same type of fever.

Although three or four days elapsed after she began to complain before she finally took to her bed, yet the disease assumed a severe form, and only terminated in convalescense during the third week.

Before convalescence was established in this case, the mother, who was now the principal nurse, was taken sick with the same disease. With her, however, it ran a mild course, and terminated favorably between the seventh and tenth days.

Here are four cases of unmistakable typhoid fever, so far as that disease can be determined by the symptoms during life. In the first no causes could be assigned, except *recency* of *residence*, and excessive exercise with protracted bathing. The second plainly originated from undue exposure to night air, and over-exertion during convalescence from another type of fever. While the third and fourth occurred in old residents during close confinement in the sick-room of the second, aided by mental anxiety and watching. If these cases did not arise from absolutely different *causes*, they certainly did from widely different circumstances. The two last are just such examples as are constantly adduced, not only to prove the existence of a specific poison, but to show also, that such poison is a true *contagion*; while the circumstances attending the first and second, though differing from each other, yet agree in being free from all traces of previous contact with the sick, or any other palpable evidences of a particular poison.

True, the advocates of a specific poison meet all such difficulties with the general assertion, that in the midst of populous communities persons come in contact with poisons and contagions without being aware of it, and consequently that inability to trace connection is no evidence of want of contact. Indeed, they would rather have us infer, that all the inhabitants, of large cities especially, are exposed to the fever poison; that many escape its action from unsusceptibility, and many more from meeting with it so diluted, by diffusion in otherwise pure air, that it is incapable of exerting its injurious effects. If this explanation would apply to the second case, by supposing that the previous attack of malarious fever had so debilitated the system, as to render it susceptible to the typhoid fever poison, how will it meet the second? It is true that his frequent and protracted bathing might debilitate the system and thereby render it more susceptible, but at the same time all the circumstances that surrounded him at home, and still more while bathing, were such as to dilute and diffuse any supposed poison to the greatest degree. It is true, that Dr. Bartlett, in the language already quoted, seems to deny that purity or any other circumstances of a local character, exercise any material influence over the fever poison; declaring both typhoid fever and cholera to be "generated as readily amidst cleanliness and purity, as amidst filth, &c." But on what part of the continent of America does Dr. Bartlett find proof for such an assertion, especially in relation to epidemic cholera? Has he, indeed, found the poison generating that disease "floating as freely in the fresh breezes" that fan his own native hills of New England, as in the "stagnant atmospheres" of Boston and New York? Has he, or any one else, seen cholera when epidemic, desolating the wide, clean, and airy streets inhabited by the wealthy in our cities, in the same ratio that it does the narrow, damp, and filthy places occupied by the poor?

For instance, has this disease ever invaded the mansions around Union Park in the city of New York, as freely and readily, as the damp and filthy habitations on Orange, Cross, Cherry, and other similar streets of the same city? The most careful examination of this subject would compel a uniform answer in the negative; and I am only surprised, that one so familiar with medical literature should have hazzarded an assertion so widely at variance with the accumulated experience of the profession at home and abroad. If the influence of local causes in determining the prevalence of typhoid fever was as clearly manifest as it is in reference to epidemic cholera, there could be no room for doubt or discussion. But it is no part of my design to enter at large upon this subject in this place; the fact that typhoid fever is frequently produced by local causes, independent of any special *fever poison*, is sustained not merely by such cases as have been detailed, but also by a great variety of evidence. First, it is the fever that prevails over the widest geographical boundaries, in climates and on soils the most diverse.

The periodical fevers are well known to be limited in their range, prevailing only in such circumscribed limits as present certain appreciable physical circumstances in reference to soil, moisture and temperature. And this has ever constituted the strongest argument in favor of attributing them to the influence of some peculiar and specific poison, generated only by a certain combination of circumstances. Typhus, too, is indisputably limited almost wholly to localities presenting a pretty uniform class of circumstances.

It is, to use the strong language of Dr. Bartlett, " in a stagnant and depraved atmosphere, rank with the thick corruptions of concentrated emanations from the living human body," that unmistakable *typhus* prevails. But typhoid fever is found to originate in every quarter of the globe, in almost every climate, and on every varity of soil. Indeed, it is *the* type to which all other types of fever tend when they run a protracted course. Thus, Dr. S. H. Dickson, in his essay on the blending of types of fever, says: " Nay, it seems to us unquestionable, that, in some sense, all the known forms of idiopathic fever predispose to *typhoid; at least all known forms of fever*, if greatly prolonged, may put on, as has been above stated, *many features which belong to the description of typhoid*, and lose all which may have been manifested during their invasion and early progress; *these latter characteristics*, however, continuing in a certain proportion to be strongly and persistently marked throughout the most indefinite protraction. This is true, not only of those already mentioned, periodical fevers of every variety, and especially in the congestive form, but of yellow fever, catarrhal fever, the consecutive fever of cholera, and the numerous pyrectic affections occupying the doubtful ground of such connection with local internal disorder as to render questionable their idiopathic character; such as gastric, mesenteric, and verminous fevers, and infantile remittents." That other fevers, when protracted, do not merely put on a typhoid state, *simulating* typhoid fever without the supposed characteristic *lesions*, but do actually become converted into the latter disease with all its local as well as general phenomena, is abundantly proved by post-mortem examinations. On this point Dr. Dickson holds the following language, viz.: "As a continued fever (typhoid) it is usually easy enough to distinguish it from bilious remittent; *yet this latter frequently puts on some of its most familiar and characteristic features:* a dry, red tongue, meteorism, muttering delirium, carphologia, coma, presenting themselves in protracted attacks; the teeth and lips being covered with dark sordes; *diarrhœa coming on with intestinal ulceration*, as seen after death." Now if this statement is true, (and we doubt whether any physician of extended experience in the treatment of fevers will deny it) we are compelled to acknowledge, that the form of fever in question may arise from a variety of causes. It would be contrary to all analogy, if not a positive anomaly in nature, to have a specific poison, *sui generis*, generated at all seasons of the year, in different climates, and almost every conceivable variety of local circumstances. That like causes, acting under like circumstances, will produce like effects, is an axiom not likely to be disputed by the careful student of nature. No one disputes the fact, that a stagnant atmosphere, loaded with animal exertions, will produce true typhus, and no one claims that the same disease will arise in the opposite circumstances, unless it is derived directly from a contagion previously generated. Equally unanimous is the opinion that a certain soil, united with the proper temperature and moisture, will generate intermittents and remittents, and very few, if any, claim that these diseases will arise, *de novo*, without such circumstances. But typhoid fever is found in prisons, emigrant ships, and jails; it is found on malarious districts, and on primitive geological formations without any of the elements of malaria; it is found at all seasons of the year; and as we have seen, it supercedes, under certain circumstances, other febrile affections of diverse origin. If these assertions are founded in truth, it necessarily follows, that typhoid fever is a condition of the system, susceptible of being induced by a variety of causes acting upon the elementary properties and functions of the system in a given direction, instead of a specific disease arising always from a single specific cause.

I am fully aware, that those writers who attribute common continued or typhoid fever to a specific poison, endeavor to avoid the force of the above facts, by claiming a distinction between a "*typhoid condition*," and what they technically call *typhoid fever.* Thus, says Dr. Flint, in the supplement to his reports on continued fever, "a typhoid *condition* is one thing, and typhoid *fever* another." And Dr. Bartlett says: "it is certainly very important, that this *typhoid state* of the system, occurring in connection with many diseases, should be distinguished from *typhoid fever.*" And he adds: "Since writing this history, I have seen a patient presenting these phenomena amongst others: prostration of strength, slight subsultus tendinum, tympanitic distension of the abdomen, diarrhœa, gurgling on pressure, a dry, red, cracked tongue, sordes on the teeth, wandering delirium, and sudamina about the neck. Here were many of the *most characteristic elements* of *typhoid fever*, but the disease was clearly and unequivocally puerpural peritonitis." Now it is certain, that just so far as the above case contained "the most characteristic elements of typhoid fever," so far, at least, it was identical with that disease. And if the case proves anything, it is simply that a typhoid type or condition of fever may be connected with peritoneal inflammation, as well as with ulcerations of the glands of the ilium. It is not merely the typhoid condition of the system that occurs in connection with fevers, primarily of a different type, but in many instances the identical intestinal lesions are present also. Dr. Dickson asserts this in a paragraph already quoted; and I have now before me portions of the ilium from two subjects, one a female, dead from well marked typhoid fever, and the other a male, attacked primarily with pneumonia, partially convalesced, but died subsequently with typhoid symptoms, and in both the glands of Peyer are extensively ulcerated in such a manner, that I think it would puzzle the most experienced morbid anatomist, to decide by an examination of the morbid specimens, which belonged to each subject. The mesenteric glands are also enlarged in both. Let not the reader infer, however, that no practical distinction is to be made between a typhoid state or fever supervening upon some previous disease, and therefore secondary, and that which commences as an idiopathic affection. On the contrary, I would take just as much care to distinguish the one class of cases from the other, as I would a diarrhœa supervening upon some previous disorder, from one occurring as the primary disease. And yet the former would be just as certainly and really a diarrhœa as the latter.

The only inferences which I would draw from the foregoing examination of facts and opinions, are: first, that intermittent and remittent fevers, being confined in their prevalence to particular, and often definitely marked, localities, are dependent on some peculiar and specific cause; second, that continued fevers, including both typhoid and typhus, are limited in their prevalence by no geographical or geological boundaries, the latter being susceptible of generation and propagation wherever human beings congregate in an impure and stagnant atmosphere, and the former wherever any cause is brought to bear on the human system capable of depressing the organic susceptibility, and thereby perverting all the primary functions, whether that *cause* consists in protracted mental anxiety and want of sleep, in insufficient food and clothing, in protracted and excessive physical exertion, in exposure to moderately impure air containing animal matter or contagious exhalations, or whether it consists in a certain exhaustion of the vital properties by the protracted continuance of previous disease. In view of these inferences it is evident that no useful classification of fevers can be founded exclusively on their exciting causes.

Much the plainest line of distinction can be drawn between those usually styled *periodical*, and those known as continued; yet, it is very evident, that the former become not unfrequently transformed into the latter. And if we may credit the common observations of our profession in the South and West, the latter not unfrequently succeeds the former upon the same soil, and to such an extent, that not a few, like Dr. Fenner, of New Orleans, attribute both types to mere modifications of the same general cause or causes. The difficulty of separating the non-eruptive contagious fevers from those which are not contagious is equally great. Whatever may be said in regard to the uniform contagiousness of true typhus, it is certain, that typhoid fever very frequently, if not generally, occurs independent of any proper contagion. So true is this, that many of the best writers express doubts in regard to its capability of being propagated by contagion in any degree. And yet, we have just the same kind of evidence, that true intestinal typhoid fever, is occasionally prŏpagated by contagion, that we have in reference to *typhus* or any other idiopathic fever. Hence we must boldly deny well observed and candidly recorded facts, or admit the conclusion, that certain fevers arising primarily from causes in no way connected with a specific contagion, may, in the course of their progress, so alter their secretions or emanations from the body of the patient, as to produce a virus capable of inducing the same disease in others. To do the first would be to impeach either the observing faculties or the veracity of our co-laborers in the science, and thereby transgress all the rules of rational inquiry; while the last is equally philosophical, and in accordance with a great number of observed facts. Those who insist most strongly on the idea, that continued fevers arise from a specific poison only, and that poison a contagious one, endeavor to avoid the force of the very obvious objection founded on the fact, that of all those having intercouse with the sick, a small portion only contract the disease, by alleging that similar escapes happen in reference to diseases notoriously contagious. Thus, Dr. Watson says in reference to this objection, "the force of this reasoning is completely broken by the well known fact, that in respect to diseases which are on all hands acknowledged to be contagious, and which are even propagable by inoculation, small-pox for example, *the same kind of exemption notoriously happens.*" Such assertions as this are not only untrue in fact, but they are well calculated to deceive the inexperienced reader. It is true, that if you bring a thousand *unprotected* persons in contact with a case of small-pox, a half dozen out of the whole number may fail to take the disease; not, however, because the room in which they were exposed was cleanly and well ventilated, but because, from some unknown cause, there was a positive want of susceptibility to the action of the poison in their systems.

(*Continued in next number.*)

